# Pathways to the senior national teams: experiences of playing-up and mixed-gender play among Swedish elite female footballers

**DOI:** 10.3389/fspor.2026.1840874

**Published:** 2026-06-22

**Authors:** Tor Söderström, Malin Andersson, Martin Holmgren, Georgios Pavlogiannis

**Affiliations:** Department of Education, Umeå School of Sport Sciences, Umeå University, Umeå, Sweden

**Keywords:** age grouping, ability grouping, soccer, talent development, talent identification

## Abstract

**Introduction:**

This study examines the significance of playing-up (participating in training and playing with older age groups) and mixed-gender play (training and playing with opposite gender) for Swedish female football players to reach the highest international level.

**Methods:**

Drawing on questionnaire data from senior female football players who play in the first league in Sweden or abroad as a professional, we examined their experiences of playing-up and mixed-gender play during their child and youth years, and its significance for being selected for the senior national teams, i.e., Swedish U23 and women's national team.

**Results and discussion:**

Findings revealed that about half of the players between age 9–12 and over 80% between age 13–15 reported experiences of training or playing with older players, showing that playing-up is a common experience among players who reach women's elite level. Regarding mixed-gender experiences, less than 25% of the participants reported that they trained or played with boys between age 9–12 and between 13 and 15 years. The findings showed further that 41% of the players had been selected for the senior national teams. Logistic regression analysis showed that experiences of playing-up or mixed-gender play between age 9–12 or 13–15 did not increase the likelihood of being selected for the senior national teams. This is one of few studies to date investigating the impact of playing-up and mixed-gender play on females' pathways to senior national team levels in football. Understanding how playing-up and mixed-gender play relates to athlete development and for attaining expertise in young adulthood can guide coaches and organizations in structuring training environments that foster sustainable development.

## Introduction

Talent development in football involves creating an environment where coaching, support, training, and match play are intentionally structured to facilitate player advancement (1). Previous research on talent development towards elite level in football shows that pathways to expert performance are complex, with evidence showing that different types of training backgrounds and environments can lead to elite level in football in adult players [e.g., (2–4)]. However, research has shown that gender biases exist and are shaped by structural and sociocultural conditions, where women experience greater variability in access to coaching, competition, and support (5, 6). Studies of elite women's academies and pathways indicate that creating appropriately challenging environments, emphasizing long-term development over early performance outcomes, and providing coherent support systems, are particularly important for sustaining development and preventing dropout among female players (6, 7). This emphasis on challenging experiences within an individual's environment has been suggested to be a key element in stimulating the development of athletes during their complex and varied journey to the elite level (8, 9). Studies examining athletes' perspectives and experiences of talent development pathways indicate that progression is influenced by various physical, technical, social, and psychological challenges [e.g., (9, 10)].

One common strategy in youth football to provide appropriate levels of challenge and foster optimal developmental settings, is to allow players that coaches deem as high-achieving to train and compete with chronologically older peers, i.e., “playing-up” (11, 12). A recent study by McEvan et al. (7) on talent development strategies in seven women's elite football clubs in Europe showed that playing-up and mixed-gender play, i.e., competing alongside boys, was a common approach among the women’s elite clubs to expose players to appropriate challenge levels. In a study comparing the developmental activities of female English footballers who achieved professional status with those who did not, Andrew et al. (2) found that around half of the participants engaged in mixed-gender play during their childhood, and about 20% during their adolescence. Despite its prevalence in North American and European youth sport programs and its claimed advantages for athlete development, empirical research explicitly investigating playing-up (12) or mixed-gender play is limited [cf. (7) on limited evidence concerning the efficacy of mixed-gender play]. Consequently, there is limited knowledge about the significance of training and playing with older athletes during the youth years, as well as engaging in mixed-gender play, for fostering development and long-term expertise among both males and females. Against this background, the current study contributes to the field of talent development by empirically examining Swedish female elite footballers' experiences of playing-up and of mixed-gender play, and the significance of these experiences for reaching the highest international level in football, i.e., the senior national teams. In doing so, the study also provides insights into the complexity and variability of athletes' developmental experiences over time.

The very few empirical studies made on playing-up and mixed-gender play have offered some initial insights into its consequences for athlete development. Gledhill's and Harwood's (13) study of developmental experiences of ten elite female youth football players found that they appreciated playing against male players between the ages of 12–14 as it enhanced their enjoyment of football and helped them to develop physical strength and perceptual skills. Regarding playing-up, the few studies made in the area show various aspects of the phenomenon. For instance, in a study with 98 male participants from an English football academy, Kelly and colleagues (12) examined factors that differentiated players who play-up from those who do not through different tests and questionnaires. Results showed that players aged under-9 to under-11 who played-up demonstrated superior ball maintenance and ability to execute accurate actions under pressure. Furthermore, players aged under-12 to under-16 were more likely to exhibit creative technical skills such as dribble completion and total touches. However, the study could not determine whether differences between those who played-up and those who did not were an effect of playing-up itself. The authors highlight the possibility that those selected by coaches to play-up may have been further ahead in terms of technical and tactical competencies, which could explain why such factors were consistent across both age phases (12).

These insights were further enriched by Goldman and colleagues (11), who interviewed Canadian youth athletes aged 13–17 (5 females, 12 males) about their perceptions of playing-up. Their findings offer a complementary perspective to the more performance-based metrics discussed by Kelly et al. (12), emphasizing the psychosocial dimensions of the experience. According to the results, playing-up was perceived as improving their sport specific skills but also, regardless of gender, that playing-up presented a duality of challenge and progress. On the one hand, they faced difficulties such as adapting to higher competition intensity and integrating socially with older teammates. On the other hand, support from coaches and teammates helped athletes navigate the initial discomfort and ultimately thrive. This aligns with the findings from Kelly et al. (12), where social factors such as peer-led play and coach-led practice were associated with playing-up, especially between the ages 12–16 years. Goldman et al. (11) also highlighted that diverse sport experiences—particularly those involving teammates with varying skill sets—were perceived as beneficial for both sport-specific and psychosocial development. This suggests that the developmental value of playing-up may not solely depend on age or skill level, but also on the contextual dynamics of the team environment.

Another widely used strategy to foster development of talents is the use of ability grouping, i.e., coaches dividing children into groups based on their current capacity (14, 15) to provide appropriate challenges. However, as with playing-up, despite its use in practice, there is little research on ability grouping in sport (16). Most research appears to be conducted in physical education contexts [e.g., (17, 18)]. Research on these grouping strategies in a school context has shown that teachers' subjective perceptions of students' abilities and how they should be divided influence access to learning opportunities and affect the level of inclusivity and equity (19). Moreover, regardless of what principles groups are formed on, students tend to be acutely aware of the differences in ability between groups (17), which may contribute to feelings of stigmatization and reinforce hierarchies among students [e.g., (17)]. In Sweden, which is the context of this study, the issue of ability grouping among children and youth is a subject of debate in which the Swedish Football Association is engaged, particularly with regard to whose interests are being served (14).

Within the Swedish context, football is by far the most common sport played by children and youth aged 7–20 years, accounting for over 40% of all organized sports participation (20, 21). However, statistics show that participation is highest among the youngest children and that there are significantly fewer girls playing football compared to boys (21). By the age of 15, there are approximately twice as many boys as girls playing football (22). Girls' participation tends to decline more sharply during adolescence (23), with girls being more likely than boys to leave organized football before reaching the senior level [(24); cf. (25)]. Mixed-gender play is allowed in Swedish football, especially under the age of 12, and it is also possible to play in mixed leagues at the children's and youth levels under specific conditions (26). Statistics on training or playing with older players, i.e., playing-up, or mixed-gender play are limited. However, Brozén and Westermark (27) report that 19%–37% of female Swedish footballers aged 13–17 play at the senior level, indicating that playing-up is common among adolescents.

Taken together, while current research indicates that developmental benefits may be associated with practices such as playing-up and mixed-gender participation during adolescence, it does not address the relationship between these strategies in youth football and progression to national or international senior elite levels. Research on playing-up or mixed-gender play for females has focused on young people and has, as Kelly et al. (12) points out concerning playing-up, not clarified if early exposure to older age groups accelerates development and contributes to long-term expertise, particularly in transitions from youth to professional levels. Moreover, despite that mixed-gender play is found to be common in girls' football and serves a talent development strategy in elite football clubs in Europe, no explicit investigation appears to have been made on how this relates to females' long-term development. To address these gaps in knowledge, this study examines Swedish female elite football players experiences of playing-up and mixed-gender play during their childhood and youth, as well as the significance of these experiences for selection to the Swedish senior national teams (U23 and women's national teams).

## Materials and methods

### Participants and procedures

This cross-sectional study uses retrospective questionnaire data from Swedish female elite football players. The classification of elite level, i.e., the two highest divisions and the transition to professional football abroad, followed the Swedish Football Association's definition of elite football. Data collection was carried out in collaboration with Elite Women's Football, an umbrella organization for the two leading leagues in Swedish women's football, which distributed information about the study by email to players in Sweden and professionals playing abroad. Given the limited response rate from second division players (*n* = 41), and to minimize bias within the scope of the available data, this study incorporates data from first-league players in Sweden as well as professional players abroad. A total of 101 players were included in the analysis, with 95 playing in the first league in Sweden and 6 playing as professionals abroad. The study utilized anonymous questionnaire data without sensitive personal information and followed the guidelines of the Swedish Research Council (28). All participants were informed about the purpose of the study and were informed that participation was voluntary and that they could choose to discontinue participation. The players who gave their consent to participate completed the online questionnaire anonymously.

However, it is difficult to know whether all players in the first league and professionals were reached with information about participating in the study. If all players were offered the opportunity to participate, the responses received correspond to approximately 22%–25% of the population of women's elite football players at highest senior level in Sweden. There are 14 teams in the first league. If we estimate an average of 25 players in each squad, plus players who play professionally abroad, this would correspond to 400–450 players in total. The average age of the players was 23.2 years (SD = 4.4). In the analyses of how playing-up and mixed-gender play relates to playing in the senior national teams, one player did not fill in data on playing-up, and players under 19 years of age were removed from the analysis as they were deemed not to be relevant for playing in these national teams. In total, 85 players of 19 years of age or older were included in the analysis of how playing-up is associated with playing in the senior national teams, i.e., Swedish U23 and women's national team.

### Measures

The results presented were part of a survey study that encompassed more than what is covered in this study. The scope of the questionnaire, and the age groups to be examined retrospectively, were established in collaboration with Elite Women's Football. The web-based questionnaire (Survey & Report) gathered information about personal information (e.g., age, birth month, and education level), previous involvement in football and in other sports, sociocultural background, experiences of playing football in different settings and present playing at elite level. The questions in the questionnaire were inspired by and based on research and questions that have been used in previous studies [e.g., (29, 30)]. In addition, the questionnaire was designed together with and reviewed by staff of Elite Women's Football. This process of formulating, designing and reviewing the questionnaire ensured the content and face validity of the questions.

For the purpose of the study, information on the players' estimation of how much they have trained and played with older players and how much they trained and played with boys between 9 and 12 years, and 13–15 years respectively was used. In Sweden, football is categorized into children's football (ages 6–12), youth football (ages 13–19), and senior football (age 20 and above). The questions were measured on a five-point Likert scale ranging from no degree at all (1) to a very high degree (5). Participation in the Swedish Football Association's national team activities was measured with a yes/no question followed by which national teams they participated in.

### Data analysis

Descriptive statistics were calculated for all study variables. Frequency counts and percentages were used to describe the categorical variable playing in senior national teams (U23 and women's national team) (yes = 1; no = 0). Mean scores with standard deviations were included for continuous factors (i.e., players estimate of playing-up and mixed-gender play during childhood and youth years).

To examine if and how the playing-up factors influence players' participation in the senior national teams (U23 and women's national team), a binary logistic regression was conducted. Unstandardized beta coefficients with standard errors, *p*-values, bootstrap 95% confidence intervals, and odds ratios (ORs) were reported. For the purposes of the regression, the eight playing-up factors (see Table 1) were reduced to four (see Table 2). This decision was based on Pearson correlation analyses, with pairs of variables showing strong correlations (|r| ≥ .70) interpreted as indicating substantial conceptual and statistical overlap. Collapsing highly correlated variables was therefore undertaken to reduce multicollinearity and improve the stability and interpretability of the regression coefficients (31).

**Table 1:** Descriptive statistics for playing-up and mixed-gender play factors (*n* = 85).

Experience	No degree at all	Fairly low degree	Neither a high nor a low degree	Fairly high degree	Very high degree	M (SD)
Age 9–12						
Trained with older players	5.8% (*n* = 5)	11.6% (*n* = 10)	26.7% (*n* = 23)	17.4% (*n* = 15)	37.2% (*n* = 32)	3.69 (1.25)
Played with older players	7% (*n* = 6)	8.1% (*n* = 7)	24.4% (*n* = 21)	19.8% (*n* = 17)	39.5% (*n* = 34)	3.78 (1.25)
Trained with boys	46.5% (*n* = 40)	17.4% (*n* = 15)	12.8% (*n* = 11)	9.3% (*n* = 8)	12.8% (*n* = 11)	2.24 (1.45)
Played with boys	59.3% (*n* = 51)	12.8% (*n* = 11)	8.1% (*n* = 7)	7% (*n* = 6)	11.6% (*n* = 10)	1.98 (1.43)
Age 13–15						
Trained with older players	1.2% (*n* = 1)	5.8% (*n* = 5)	10.5% (*n* = 9)	26.7% (*n* = 23)	54.7% (*n* = 47)	4.29 (.96)
Played with older players	3.5% (*n* = 3)	3.5% (*n* = 3)	10.5% (*n* = 9)	27.9% (*n* = 24)	53.5% (*n* = 46)	4.26 (1.02)
Trained with boys	55.8% (*n* = 48)	7% (*n* = 6)	12.8% (*n* = 11)	14% (*n* = 12)	9.3% (*n* = 8)	2.13 (1.45)
Played with boys	70.9% (*n* = 61)	5.8% (*n* = 5)	9.3% (*n* = 8)	4.7% (*n* = 4)	8.1% (*n* = 7)	1.72 (1.29)

**Table 2:** Descriptive statistics for playing-up and mixed-gender play by selection to senior national teams (U23 and women's national team) (*n* = 85).

Experience	Not Selected (n 50) M (SD)	Selected (n 35) M (SD)	Total M (SD)
Age 9–12			
Trained and played with older players	3.88 (1.16)	3.48 (1.32)	3.72 (1.28)
Trained and played with boys	1.93 (1.30)	2.28 (1.51)	2.16 (1.40)
Age 13–15			
Trained and played with older players	4.32 (.96)	4.20 (1.02)	4.32 (.94)
Trained and played with boys*	1.67 (1.19)	2.20 (1.30)	1.98 (1.32)

* Represents statistical differences, *p* < .05.

Specifically, for ages 9–12, the items “trained with older players” and “played matches with older players” were very strongly correlated (*r* = .962, *p* < .001) and were combined into a single factor labelled “trained and played with older players (ages 9–12)”. Similarly, the items “trained with boys” and “played matches with boys” showed a strong correlation (*r* = .874, *p* < .001) and were combined into a single factor. The same pattern was observed for ages 13–15, where “trained with older players” and “played matches with older players” were highly correlated (*r* = .942, *p* < .001), as were “trained with boys” and “played matches with boys” (*r* = .763, *p* < .001). The rest of the correlations were moderate and low and were therefore not considered indicative of problematic overlap. The four collapsed playing-up factors included in the regression are presented in detail in Table 2.

The dependent variable of the regression was if they have been selected to the senior national teams (U23 and women's national team).

Prior to analysis, regression assumptions were tested. The continuous variables fulfilled the requirement for logistic regression. The data were screened to ensure that all observations were independent. No repeated measures or clustering were present. No significant outliers were identified. Multicollinearity was assessed using tolerance and variance inflation factor (VIF) values from a linear regression model with the predictors. All tolerance values exceeded .10, and all VIF values were below 10, indicating that multicollinearity was not a concern (32). The linearity of the logit was tested for all continuous predictors by including interaction terms between each predictor and its natural logarithm. None of the interaction terms were statistically significant (*p* > .05), indicating the assumption of linearity in the logit was met (33). Independent samples t-tests were subsequently performed to assess differences in mean scores on the four collapsed playing-up factors between athletes selected for the senior national teams (U23 and women's national team) and those who were not selected. The effect size was calculated using Cohen's d. The assumption of homogeneity of variances was assessed using Levene's test, and Welch's *t*-test was applied where this assumption was violated.

## Results

Table 1 presents descriptive statistics for the extent to which the players trained and played games with older players and/or boys between age 9–15. Table 1 highlights that the players reported that they have to a fairly or very high degree trained or played with older players between age 9–12 (54.6% vs. 59.3%) and between age 13–15 (81.4% vs. 81.4%). Mean scores for training and playing with older players between age 9–12 were above its scale mid-point whereas mean scores between age 13–15 were well above its scale mid-point. Less than 25% of the players reported that they trained or played with boys between age 9–12 (21.1 vs. 18.6%) and between age 13–15 (23.3 vs. 12.8%). Mean scores for training and playing with boy players between age 9–15 were below its scale mid-point.

The results show further that 35 (41%) of 85 players of 19 years of age or older have participated in the senior national teams (U23 and women's national team). Table 2 shows descriptives for the four collapsed playing-up and mixed-gender factors between the age of 9 to 15 for players that participated and those who did not participate in senior national teams.

Table 2 shows that participants selected to the national teams compared to those not selected report slightly lower levels of playing and training with older players between ages 9–12 and 13–15 but higher levels of playing and training with boys during both 9–12 and 13–15 age periods. Selected players reported training and playing with boys to a significantly higher degree between ages 13–15 than non-selected players [*t*(82) = −1.920, *p* < .05, *d* = 1.23].

### Binary logistic regression analysis

A binary logistic regression was conducted to examine how the playing-up factors influence players' selection to the senior national teams (U23 and women's national team). The overall model was not statistically significant, *χ*²(4) = 6.63, *p* = .157, suggesting the predictors did not significantly improve prediction over the null model. The model explained between 7.6% (Cox & Snell R^2^) and 10.2% (Nagelkerke R²) of the variance in team selection and correctly classified 60.7% of cases, a modest increase from 58.3% accuracy of the null model. The low pseudo-R² values indicate limited explanatory power of the model, suggesting that playing-up experiences alone account for only a small proportion of variance in senior national teams (U23 and women's national team) selection, a pattern consistent with the multifactorial nature of elite sport selection processes.

Table 3 presents the findings of the binary logistic regression analysis examining the impact of playing-up and mixed-gender factors on selection to the senior national teams (U23 and women's national team).

**Table 3:** Findings from binary logistic regression analysis.

Predictors	B	SE	OR—Exp(B)	95% CI for Exp(B)	*p*
Between 9 and 12 I played and trained with older players	–0.368	0.223	0.692	0.447–1.072	.099
Between 9 and 12 I played and trained with boys	0.058	0.186	1.059	0.735–1.526	.757
Between 13 and 15 I played and trained with older players	0.159	0.283	1.173	0.674–2.042	.573
Between 13 and 15 I played and trained with boys	0.356	0.213	1.428	0.941–2.166	.094
Constant	–0.464	1.155	0.628	–	.688

B, unstandardized logistic regression coefficient; SE, standard error; OR, odds ratio [Exp(B)]; 95% CI, 95% confidence interval for the odds ratio. None of the predictors were statistically significant at *p* < .05.

Table 3 shows that playing-up or mixed-gender play between ages 9–12 or 13–15 did not significantly predict selection to the senior national teams (U23 and women's national team). None of the playing-up or mixed-gender variables reached statistical significance at the *p* < .05 level, suggesting that early experiences of playing-up or mixed-gender play may not be critical factors in progressing to senior national teams.

## Discussion

The purpose of this study was to investigate female elite football players experiences of playing-up and mixed-gender play during their child and youth years and its significance for being selected to the Swedish senior national teams. Overall, two major patterns emerged from the results of this study. The first pattern showed that it is common among Swedish elite female football players to have trained and played with older players, while less than 25% of the participants reported that they trained or played with boys. The second pattern highlighted that experiences of playing-up or of mixed-gender play did not significantly increase the likelihood of being selected to the U23 and women's national team. In the next sections, we discuss the study's findings in greater detail.

### Playing-up and mixed-gender play

The results show that playing-up has been part of the development environment for about half of the players aged 9–12 and for over 80% of those aged 13–15. Regarding the players' experiences of mixed-gender training and play, the results show that players have less experience of this compared to playing-up. While mixed-gender play is a common approach in women's elite football clubs (7) and experienced by around half of English female elite players during childhood (2), the Swedish female elite footballers in this study reported less frequent participation in such activities. These divergent results raise questions about potential cultural or structural differences in talent development environments, questions that need to be further investigated in future research.

Although the findings show that playing-up is a common experience, especially during adolescence and what Côté and Vierimaa (34) terms the specialization phase, this study cannot determine how and in what way playing-up has challenged or influenced players' development for reaching the senior elite level. However, it can be concluded that the players’ experiences of playing-up are consistent with the idea that players are often exposed to progressively more challenging environments during adolescence [(11), see also (12) on adolescent male participants from an English football academy]. The fact that over 80% of the players included in this study have trained and played with older players between the ages of 13–15 may not only be a consequence of a deliberate “playing-up strategy” employed by coaches or clubs to expose players to appropriate challenge levels, fostering development and long-term expertise [cf. (7)]. It could also be a consequence of that there are many fewer girls than boys playing football in Sweden, and girls having higher drop-out rates (21–23), which forces clubs to fill up squads with younger players to maintain a sufficient amount of players [cf. (27) on the amount of girls playing at the senior level]. The selection of players to fill these squads is likely based on, as Kelly et al. points out in their study (12), coaches' judgements of players who possess the football skills needed to play with older players.

### Selection for the U23-senior national team

The second pattern identified in this study, as shown by the logistic regression analysis in Table 3, is that that neither training or playing matches with older players nor training or playing matches with boys both between ages 9–12 and 13–15 significantly increased the likelihood of being selected for the U23 and women's national team. While previous research has shown that boys who play up demonstrate more advanced football skills (12) and that youth athletes perceive playing-up as a way of improving their skills (11), this study shows that even though playing-up is a common experience among Swedish elite-level female football players, it does not increase the likelihood of making it to the senior national teams (U23 and women's national teams). The analysis of players selected for senior national teams and those not selected, shows no significant difference between the groups related to experiences of playing-up (see Table 2). In fact, the descriptive statistics show that the players selected for national teams to a somewhat lesser extent engaged in playing-up between age 9–12 while it was at about the same level between 13 and 15 years (see Table 2).

Furthermore, although studies have found that female elite youth players report benefits from mixed-gender play [e.g., physical and perceptual development (13)], and that it is common during childhood among female professional players (2), the logistic regression analysis (Table 3) shows that mixed-gender training and play did not increase the likelihood of selection to senior national teams. However, the descriptive statistics (Table 2) shows that those who have been selected to the senior national teams have engaged in mixed-gender training and play to a greater extent, especially between the ages of 13–15. This difference between selected and non-selected players during ages 13–15 was both statistically significant and associated with a large effect size (*d* = 1.23), indicating a substantial group difference. This suggests that engagement in mixed-gender environments during adolescence may be associated with progression to the senior national team level. The reason why this effect occurs between the ages of 13 and 15 and not between the ages of 9 and 12, as shown in Table 2, can be understood in different ways.

On the one hand, it may be related to growth and maturation, which can influence the degree of challenge that girls experience in mixed-gender settings. Between the ages of 9 and 12, there are fewer differences between boys and girls in terms of physical qualities (35), whereby a mixed-gender environment does not pose any significant challenges for girls. However, between the ages 13–15, the improvements in physical qualities that boys experience in connection with their pubertal development [cf. (35)] most likely lead to an increase in speed, power and intensity in the training environment. Thus, girls need to adapt to these changes in training and match environments after the age of 13, which may provide developmental challenges that contribute to their physical, perceptual and decision-making capacities [cf. (13)].

On the other hand, the difference found between selected and non-selected players during ages 13–15 may not be a consequence of a more challenging and developmental environment, but rather a reflection of a selection process. It could be that coaches deemed that those girls who reported training (≈20%) and playing (≈13%) with boys to a greater extent, had the specific football skills and physical qualities required to keep up with the increased speed, power and intensity [cf. (12) on selection for playing-up]. Girls demonstrating these skills are probably more likely to be selected for mixed-gender training environments and in this sense, mixed-gender play can be seen as more of an indicator of who can cope with the increased demands, rather than as a factor that affects girls’ development.

The potential explanations proposed here regarding the association between mixed-gender training and play at ages of 13 and 15 and subsequent progression to senior national teams, should be regarded as hypothetical and speculative in nature. The observed difference between selected and non-selected players at these ages should be interpreted with caution, as mixed-gender play was not a significant predictor in the logistic regression analysis. While the difference between groups is significant in the univariate analysis (Table 2), this does not provide sufficient evidence to reliably determine how mixed-gender training and play predict selection to the U23 and women's national team.

### Concluding remarks

This study has addressed a specific gap in the literature and provide important insights about the impact of playing-up and mixed-gender play within the context of female football. Previous studies have shown that both playing-up and mixed-gender play is common, and that they can have positive effects on young people's sports development (11, 12). What has not been investigated previously, and what this study has contributed knowledge about, is the relationship between these strategies in youth football, and how they influence progression towards senior national teams. The results thus raise questions about the extent to which talent development strategies that emphasize developmental settings, where playing-up and mixed-gender play is cherished, especially during childhood, actually hold the importance they are assumed to have for progression towards elite sport levels in adulthood. At the same time, the large effect size observed for mixed-gender play during ages 13–15 suggests that certain developmental experiences during adolescence may still be meaningfully associated with elite progression, even if they do not serve as independent predictors.

Although playing-up and mixed-gender play has been in focus, the findings of the study also raise questions about what impact the used and debated strategy of ability grouping in child football in Sweden has in the long term (14, 15). The arguments behind this are that those players who have progressed further need challenges to develop. Most likely the “better” children will appreciate this and gain motivation and energy while others, as Hastie et al. (17) highlight as a consequence of ability grouping in a school context, may feel stigmatized. Playing in a group with the best players before the age of 13 will most likely advance football specific skills [cf. (12)]. However, as with playing-up and mixed-gender play, knowledge about its significance of fostering development and long-term expertise beyond childhood and adolescence is currently underdeveloped. Further research is required to clarify the hypothetical reasoning discussed and to obtain better data to improve understanding of the long-term implications of current player development strategies. Research should also shed light on the potential cultural and structural differences raised by this study.

### Limitations and implications for future research

As only part of all the elite female football players in Sweden was investigated, no claims can be made that the findings can be generalized to the entire population of elite female football players in Sweden. The relatively modest response rate (22%–25%) also raises the possibility of self-selection bias, as players who chose to participate may differ systematically from non-respondents, for example in terms of interest in development pathways or experiences of playing-up and mixed-gender play. This limitation should be considered when interpreting the findings. In addition, given that participants were elite players reflecting on early developmental experiences, their accounts may be influenced by subsequent career outcomes or dominant narratives of athlete development. Although retrospective methods are commonly used in talent development research, this limitation should be acknowledged when interpreting the precision of reported experiences.

Finally, future studies involving a larger and more representative sample of elite female football players may provide more robust evidence and help resolve the analytical uncertainties identified, thereby offering clearer insight into how both playing-up and participating in mixed-gender environments relate to progression to senior national teams. In addition, larger samples, including elite and non-elite players, men and women, can more clearly provide greater accuracy for more precisely distinguishing whether and to what extent how playing-up and mixed-gender play in childhood and adolescence is associated with progression to national and international senior elite levels. In such future research, cross-sectional and longitudinal designs with a combination of survey and interview data that provide more granular information about both the extent and meaningfulness of playing-up and mixed-gender play, are needed. It is, however, important to emphasize the importance of interpreting such relationships within the complex and multifactorial nature of talent development pathways (2–4). This would provide more reliable and credible data on the impact of playing-up and mixed-gender play on athlete development.

## Data Availability

The datasets presented in this article are not readily available because of the specific group of participants included. Inquiries about the data included in the study can be directed to the corresponding author. Requests to access the datasets should be directed to tor.soderstrom@umu.se.
